# GLP-1 and GLP-1/GIP receptor agonist medication use and self-reported outcomes in individuals with lipedema: Results from a large online survey

**DOI:** 10.1016/j.obpill.2026.100316

**Published:** 2026-08-14

**Authors:** Ashok Srinivasan, Jonathan Kartt, Felicitie Daftuar, Laura D. Harmacek, Elena Samouhos, Stephanie Galia, Stacey Heil, Erin Clark, Courtney Mascio, Jesse C. Cochrane

**Affiliations:** Lipedema Foundation, 450 Lexington Ave, New York, NY, 10017, USA

**Keywords:** GLP-1 receptor agonists, Incretin therapy, Lipedema, Patient-reported outcomes, PROMIS, Survey

## Abstract

**Background:**

Lipedema is a chronic medical condition primarily affecting women, characterized by bilateral, symmetrical, disproportionate fat deposition in the lower limbs, and sometimes arms and lower trunk. Pharmacological interventions remain limited, but glucagon-like peptide-1 receptor agonists (GLP-1 RAs) and dual GLP-1/glucose-dependent insulinotropic polypeptide receptor agonists (GLP-1/GIP RAs) could provide potential therapeutic benefit due to their effects on weight, metabolism, pain, and inflammation.

**Methods:**

This was a cross-sectional, patient-reported online survey study comparing self-reported lipedema symptom severity, medication use history, and physical and mental health outcomes across three groups of women with lipedema stratified by GLP-1/GIP receptor agonist medication use status (current users, discontinued users, and never-users). The survey captured demographics, lipedema characteristics, medication use history, and intensity of lipedema symptoms. Additionally, the Patient-Reported Outcomes Measurement Information System (PROMIS) Global Health-10 (PROMIS-10) instrument was used to assess physical and mental health. Analyses compared current users, discontinued users, and never-users of GLP-1/GIP RA medications.

**Results:**

Of 2852 respondents, 2719 met inclusion criteria for analysis. Most participants (99.7%) identified as female, with a mean age of 52.4 years. Approximately 55% were current GLP-1/GIP RA users, most commonly taking tirzepatide. The primary reported reason for medication use was weight management (67.2%), followed by lipedema symptom management (19.3%). PROMIS-10 scores demonstrated higher physical and mental health ratings among current users compared with never-users (median Global Physical Health (GPH): 42.3 vs. 39.1; Global Mental Health (GMH): 44.9 vs. 40.8). Participants recalled improvements in both general health and lipedema symptoms after starting medication. Symptom severity scores for pain, swelling, and functional limitation were consistently lower among current users.

**Conclusions:**

Self-reported participant responses indicated improved health and symptom severity while using GLP-1/GIP RA medication. These findings support further investigation of GLP-1/GIP RA medications as potential therapeutic options for lipedema.

## Graphical abstract

**Figure:**
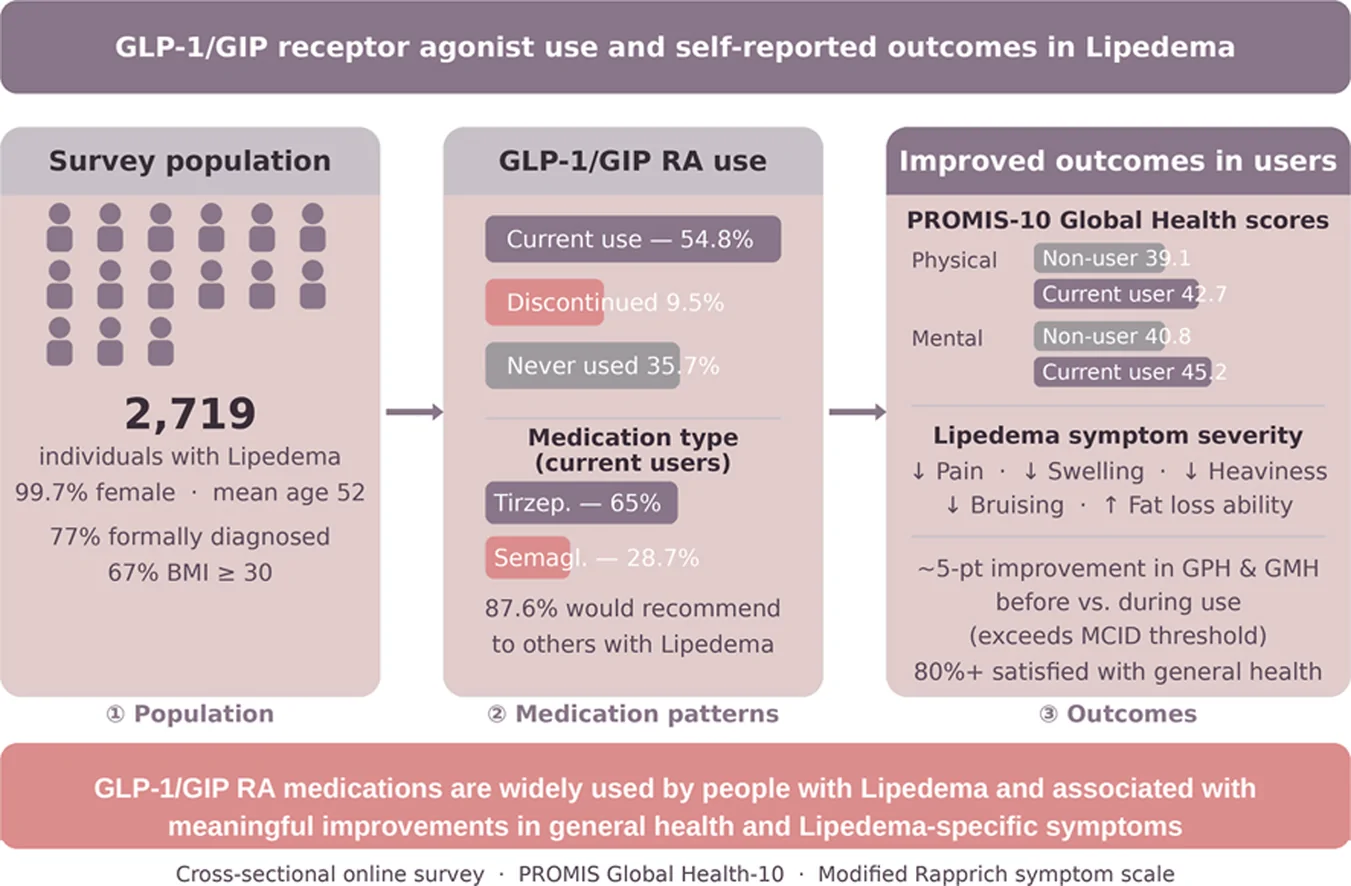

## 1 Introduction

Lipedema is a chronic adipose tissue disorder predominantly affecting women, characterized by bilateral and symmetrical enlargement of the lower and/or upper extremities, pain, easy bruising, and progressive functional limitations. The lower trunk (hips, buttocks, and lower abdomen) may also be affected [1]. Unlike typical obesity, lipedema fat is largely resistant to dietary modification and physical activity. Patients frequently report minimal or no reduction in affected tissue despite sustained caloric restriction and exercise, which can contribute to significant psychological distress and delayed diagnosis [2]. The pathophysiology of lipedema involves chronic low-grade inflammation, with evidence of elevated pro-inflammatory cytokines, adipose tissue macrophage infiltration, and microvascular dysfunction that may drive both the progressive enlargement and the pain characteristic of the condition [3]. The condition is often misdiagnosed as obesity, and its treatment options are limited, primarily consisting of conservative management (including compression therapy, manual lymphatic drainage) and liposuction [4].

Glucagon-like peptide-1 receptor agonists (GLP-1 RAs) and dual GLP-1/GIP receptor agonists (GLP-1/GIP RAs) are widely prescribed for obesity and type 2 diabetes and have shown efficacy in promoting weight loss, reducing systemic inflammation, and improving metabolic parameters [5,6]. Given that lipedema is often associated with increased adiposity and inflammation, these medications have gained interest among patients and clinicians as potential adjunctive therapies.

The aim of this study was to characterize the use of incretin mimetics (GLP-1 or GLP-1/GIP RA medications) in a large sample of individuals with lipedema, to examine self-reported outcomes related to general health and lipedema symptom severity, and to assess patient satisfaction with these medications.

## 2 Methods

### 2.1 Study design and participants

This was a cross-sectional, patient-reported online survey study comparing self-reported lipedema symptom severity, medication use history, and physical and mental health outcomes (via the Patient-Reported Outcomes Measurement Information System (PROMIS) Global Health-10 instrument) across three groups of women with lipedema stratified by their GLP-1/GIP receptor agonist medication use status (current users, discontinued users, and never-users). Institutional Review Board (IRB) approval for the study was obtained through North Star Non-Profit Research Ethics Review Board, ensuring that all study procedures complied with ethical standards for research involving human subjects. Participants provided informed consent prior to beginning the survey, and all responses were collected anonymously to protect confidentiality.

We conducted a cross-sectional, anonymous online survey using a secure, validated platform for questionnaire development and analysis (SurveyMonkey, San Mateo, CA, United States). The survey was launched at the Fat Disorders Resource Society (FDRS) Meeting (Atlanta, GA) on March 21, 2025 and closed on May 3, 2025. The survey was disseminated in person at FDRS as well as through multiple other channels to reach individuals with lipedema. Recruitment avenues included the Lipedema Foundation newsletter, social media platforms (e.g., Facebook, Instagram, and relevant online support communities), and registry email listserv.

### 2.2 Inclusion and exclusion criteria

Participants were included if they were ≥18 years of age, provided consent, and responded “Yes” to having a formal lipedema diagnosis by a medical professional or to suspecting they have lipedema. Respondents who did not answer the question on GLP-1/GIP RA medication use were excluded.

### 2.3 Survey content

The survey collected:

● **Demographics:** Age, sex, Boday Mass Index (BMI) (calculated from self-reported height and weight)
● **Diagnosis status:** Formal Diagnosis (“Yes, I have been formally diagnosed by a medical professional”), Self Diagnosis (“I have not been formally diagnosed by a medical professional, but I suspect I have Lipedema”) and “No”
● **Lipedema profile:** Age of symptom onset, Stage (Stage 1, 2, 3, 4 (lipo-lymphedema), Unsure, Unknown), Affected areas (Hips, Buttocks, Upper legs, Lower legs, Upper arms, Abdomen, Other, Multiple)
● **Medication use:** Any GLP-1 (Yes to “Have you used (or are you currently using) a GLP-1 receptor agonist medication or a GLP-1/GIP dual agonist medication?”), No GLP-1 (No to “Have you used (or are you currently using) a GLP-1 receptor agonist medication or a GLP-1/GIP dual agonist medication?”), Current Users (Any GLP-1 and Yes to “Are you currently taking GLP-1 medication?”), Discontinued Users (Any GLP-1 and No to “Are you currently taking GLP-1 medication?”)
● **Medication profile:** Formulation of GLP-1/GIP RA medications (semaglutide, tirzepatide, other (dulaglutide, exenatide, lixisenatide [discontinued in the United States and the European Union], retatrutide [not FDA approved]), Multiple (any combination)), Type (branded vs. compounded), and Reason for use (Lipedema symptom relief, Weight management (overweight/obesity), Type 2 diabetes or prediabetes management, Other)
● **Satisfaction measures:** Perceived effectiveness on General Health (Very satisfied, Somewhat satisfied, Neutral, Somewhat dissatisfied, Very dissatisfied, Unknown), Perceived effectiveness on Lipedema Symptoms (Very satisfied, Somewhat satisfied, Neutral, Somewhat dissatisfied, Very dissatisfied, Unknown), Likelihood of recommending them (Definitely yes, Probably yes, Unsure, Probably no, Definitely no, Unknown)
● **Patient-reported outcomes:** Patient-Reported Outcomes Measurement Information System (PROMIS) Global Health (PROMIS-10) questionnaire (Global Physical Health [GPH], Global Mental Health [GMH] [[7], [8], [9]]). The PROMIS-10 is a validated 10-item instrument that generates two summary scores: GPH and GMH. Both scores are standardized to the U.S. general population with a mean of 50 and a standard deviation (SD) of 10, where a score of 50 represents average health for a typical American adult. Scores at or below 40 indicate functioning approximately one standard deviation below the population mean, reflecting substantially impaired physical or mental health. Higher scores indicate better health across both dimensions.
● **Lipedema symptoms:** Modified from Rapprich et al. [10]. Participants were asked to rate the level of pain or discomfort on a scale from 0 to 10: Do you experience pain or tenderness in the areas affected by Lipedema?, Do you experience a feeling of warmth or coldness in the areas affected by Lipedema?, Do you experience frequent bruising in the areas affected by Lipedema?, Do you experience any swelling in the areas affected by Lipedema?, Do you experience the feeling of tired or heavy legs?, Do you have any nodules or lipomas in the areas affected by Lipedema?, Is the tissue in the areas affected by Lipedema fibrotic?, Are you able to lose fat in the areas affected by Lipedema?, Do you experience limitations in your ability to perform daily tasks or move around when limited by Lipedema (e.g., heaviness in the legs)?, Do you experience a general sense of forgetfulness, i.e. difficulty remembering names, dates or events?, Do you experience any difficulty to focus on tasks or maintain your train of thought during conversation?

### 2.4 Statistical analysis

Descriptive statistics were calculated for demographics, lipedema characteristics, and medication use patterns. PROMIS-10 scores and symptom ratings were compared across three groups: current users, discontinued users, and never-users. Within-subject changes in self-reported health before and after medication initiation were examined among current and discontinued users. Group comparisons were performed using nonparametric Wilcoxon rank-sum tests, implemented in R (version 4.4.3; R Core Team, Vienna, Austria) and R Studio (2025.09.0 + 387; Posit Software, PBC) with the rstatix package. P-values were adjusted for multiple comparisons using the Bonferroni method, and significance levels were annotated based on adjusted p-values.

All analyses were conducted in R (version 4.4.3) using the tidyverse and rstatix packages. Data visualization was performed with ggplot2, with figure layouts refined using patchwork, and results annotated using ggpubr functions to integrate statistical test outputs into plots. Missing data were handled by excluding unanswered items from analysis on a pairwise basis; thus, the sample size (n) is reported for each analysis and may vary slightly across outcomes. Figures were designed to highlight differences across groups and within-subject changes, with consistent scales, colors, and themes to facilitate interpretability.

## 3 Results

### 3.1 General characteristics of survey participants

**Fig. 1:**
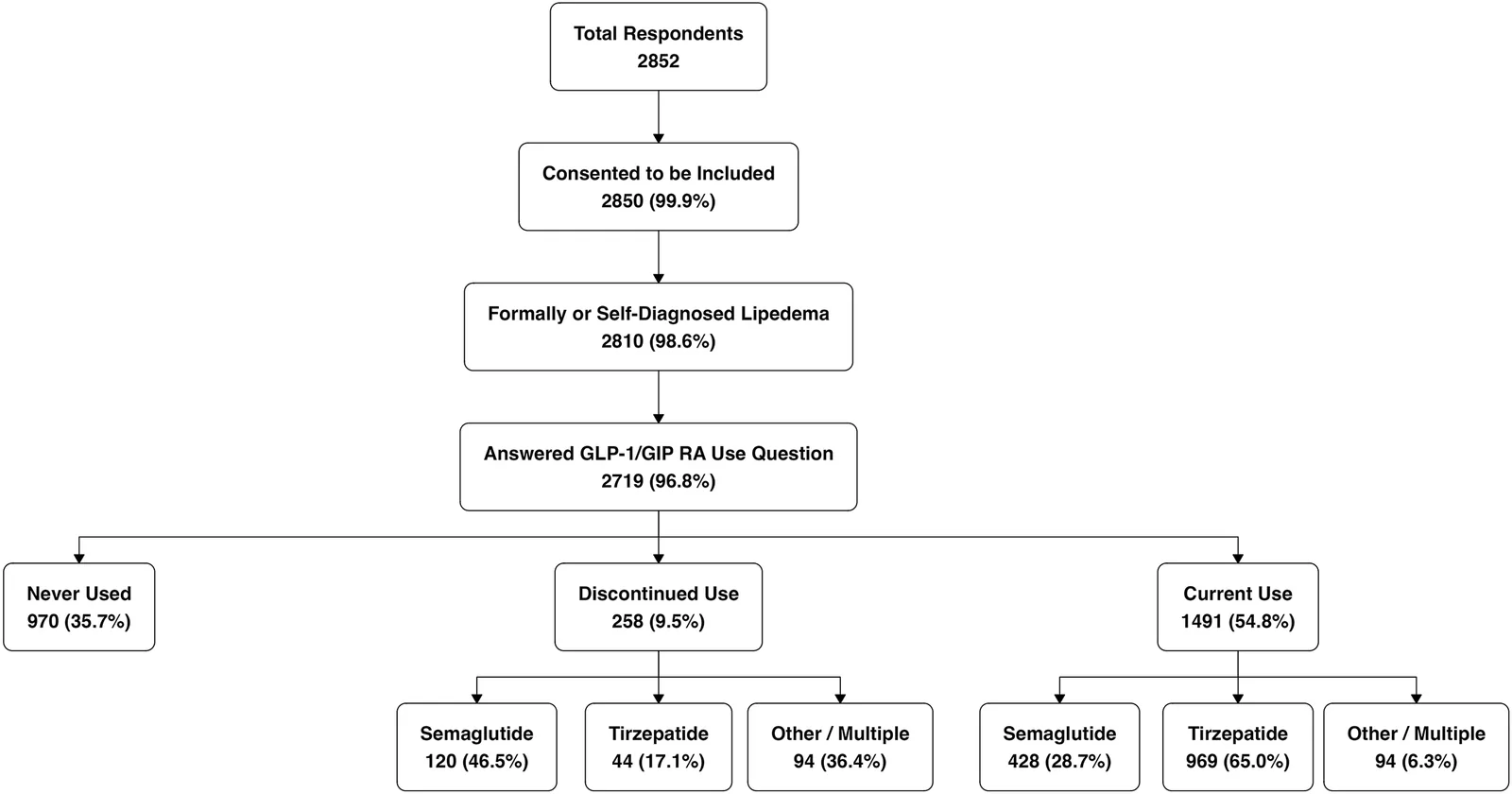
Flowchart of Participant inclusion and GLP-1 receptor agonist use in survey respondents.

A total of 2852 individuals responded to the survey with 2719 people meeting all the inclusion criteria (Fig. 1). This group of 2719 represents the final analytical sample. Of these, 2710 (99.7%) identified as female, with a mean age of 52.4 years (Table 1). Most participants (67.0%) reported a self-assessed BMI in the obesity range (BMI ≥30). A formal lipedema diagnosis was reported by 2103 individuals (77.3%) with the average age of symptom onset observed in their early 20s. The majority (68.1%) of respondents reported having Stage 2 or Stage 3 lipedema [11]. Most people (over 97%) reported that lipedema signs and symptoms affected multiple body areas; almost all of these included their upper legs (96.2%) (Table 1).

**Table 1:** **Analysis of Demographic Characteristics by GLP-1/GIP Receptor Agonist Use Status** This table presents demographic and clinical characteristics of study participants (n = 2719) stratified by medication use status: Never Used (n = 970), Discontinued Use (n = 258), and Current Use (n = 1491). Data are presented as frequencies with percentages for categorical variables and means with standard deviations (SD) for continuous variables.

Variable	Overall	Current Use	Discontinued Use	Never Used
**Total Respondents**				

N	2719	1491	258	970
**Sex**				

Female	2710 (99.7%)	1486 (99.7%)	258 (100.0%)	966 (99.6%)
Male	8 (0.3%)	4 (0.3%)		4 (0.4%)
Other	1 (0.0%)	1 (0.1%)		
**Age (Years)**				

Mean (SD)	52.4 (11.6)	50.8 (10.5)	52.0 (11.3)	54.8 (12.7)
**Height (Inches)**				

Mean (SD)	64.8 (2.7)	65.0 (2.7)	64.8 (2.9)	64.6 (2.7)
**Weight (Pounds)**				

Mean (SD)	215.5 (64.7)	210.3 (62.6)	237.8 (73.8)	217.5 (63.9)
**BMI (Calculated)**				

Healthy Weight	305 (11.2%)	203 (13.6%)	11 (4.3%)	91 (9.4%)
Obesity	1822 (67.0%)	950 (63.7%)	204 (79.1%)	668 (68.9%)
Overweight	525 (19.3%)	311 (20.9%)	40 (15.5%)	174 (17.9%)
Underweight	9 (0.3%)	3 (0.2%)	1 (0.4%)	5 (0.5%)
Unknown	58 (2.1%)	24 (1.6%)	2 (0.8%)	32 (3.3%)
**Diagnosis Status**				

Formal Diagnosis	2103 (77.3%)	1180 (79.1%)	200 (77.5%)	723 (74.5%)
Self Diagnosis	616 (22.7%)	311 (20.9%)	58 (22.5%)	247 (25.5%)
**Age of Symptom Onset**				

Mean (SD)	22.0 (14.1)	21.0 (12.6)	22.8 (14.6)	23.5 (16.1)
**Diagnosed Stage of Lipedema**				

Stage 1	207 (7.6%)	104 (7.0%)	13 (5.0%)	90 (9.3%)
Stage 2	1136 (41.8%)	655 (43.9%)	98 (38.0%)	383 (39.5%)
Stage 3	715 (26.3%)	403 (27.0%)	74 (28.7%)	238 (24.5%)
Stage 4 (lipo-lymphedema)	343 (12.6%)	172 (11.5%)	46 (17.8%)	125 (12.9%)
Unknown	9 (0.3%)	5 (0.3%)		4 (0.4%)
Unsure	309 (11.4%)	152 (10.2%)	27 (10.5%)	130 (13.4%)
**Body Areas Affected by Lipedema**				

Hips	1895 (69.7%)	1052 (70.6%)	175 (67.8%)	668 (68.9%)
Buttocks	1844 (67.8%)	1032 (69.2%)	176 (68.2%)	636 (65.6%)
Upper legs	2617 (96.2%)	1438 (96.4%)	247 (95.7%)	932 (96.1%)
Lower legs	2197 (80.8%)	1221 (81.9%)	211 (81.8%)	765 (78.9%)
Upper arms	2176 (80.0%)	1215 (81.5%)	221 (85.7%)	740 (76.3%)
Lower arms	744 (27.4%)	423 (28.4%)	93 (36.0%)	228 (23.5%)
Abdomen	1377 (50.6%)	768 (51.5%)	149 (57.8%)	460 (47.4%)
Other	180 (6.6%)	81 (5.4%)	26 (10.1%)	73 (7.5%)
Multiple	2654 (97.6%)	1461 (98.0%)	252 (97.7%)	941 (97.0%)

NOTE: Denominator based on number of non-missing responses.

Among the analytical sample, 1491 individuals reported currently using incretin mimetics (Current Use), 258 had used them in the past but were no longer taking them (Discontinued Use) and 970 reported never having used GLP-1/GIP RA medications (Never Used). Among those currently using incretin mimetics, tirzepatide was the most common (65.0% of respondents) while those who had discontinued use reported semaglutide (46.5% of respondents) as being the most prevalent (Fig. 1).

### 3.2 GLP-1/GIP RA medication use

Over 85% of the respondents who reported ever using any GLP-1/GIP RA medication are currently using these medications. Most people (66.0%) have used incretin mimetic medications for less than a year (among both current users and those who stopped). Approximately 65% (1013) of incretin mimetic users (both current and discontinued) reported taking either a branded or compounded version of tirzepatide with the majority (76%) using branded versions (Table 2).

**Table 2:** **Analysis of GLP-1/GIP Receptor Agonist Medications by Medication Type** This table presents characteristics of participants who have ever used GLP-1/GIP RA medications (n = 1749), stratified by medication type: Any GLP-1 (all users), Semaglutide (n = 548), Tirzepatide (n = 1013), and Other/Multiple medications (n = 188). Data are presented as frequencies with percentages for categorical variables and means with standard deviations (SD) for continuous variables.

Variable	Any GLP-1	Semaglutide	Tirzepatide	Other/Multiple
**Total Respondents**				

N	1749	548	1013	188
**Formulation of GLP-1/GIP RA Medication**				

Branded version	1263 (72.2%)	378 (69.0%)	769 (75.9%)	116 (61.7%)
Compounded version	417 (23.8%)	159 (29.0%)	222 (21.9%)	36 (19.1%)
Unknown	69 (3.9%)	11 (2.0%)	22 (2.2%)	36 (19.1%)
**Time on GLP-1/GIP RA Medication (in Days)**				

Mean (SD)	321.0 (354.3)	351.5 (369.1)	292.3 (296.3)	405.2 (573.8)
**Time on GLP-1/GIP RA Medication**				

Less than a year	1155 (66.0%)	343 (62.6%)	716 (70.7%)	96 (51.1%)
1–2 years	32 (1.8%)	12 (2.2%)	6 (0.6%)	14 (7.4%)
2–3 years	9 (0.5%)	5 (0.9%)	1 (0.1%)	3 (1.6%)
More than 3 years	44 (2.5%)	19 (3.5%)	17 (1.7%)	8 (4.3%)
Unknown	70 (4.0%)	13 (2.4%)	16 (1.6%)	41 (21.8%)
**Satisfaction with GLP-1/GIP RA Medication on General Health**				

Very satisfied	905 (51.7%)	237 (43.2%)	619 (61.1%)	49 (26.1%)
Somewhat satisfied	454 (26.0%)	170 (31.0%)	238 (23.5%)	46 (24.5%)
Neutral	124 (7.1%)	49 (8.9%)	55 (5.4%)	20 (10.6%)
Somewhat dissatisfied	79 (4.5%)	36 (6.6%)	29 (2.9%)	14 (7.4%)
Very dissatisfied	145 (8.3%)	53 (9.7%)	63 (6.2%)	29 (15.4%)
Unknown	42 (2.4%)	3 (0.5%)	9 (0.9%)	30 (16.0%)
**Satisfaction with GLP-1/GIP RA Medication on Lipedema Symptoms**				

Very satisfied	621 (35.5%)	162 (29.6%)	419 (41.4%)	40 (21.3%)
Somewhat satisfied	529 (30.2%)	181 (33.0%)	312 (30.8%)	36 (19.1%)
Neutral	327 (18.7%)	114 (20.8%)	174 (17.2%)	39 (20.7%)
Somewhat dissatisfied	101 (5.8%)	37 (6.8%)	48 (4.7%)	16 (8.5%)
Very dissatisfied	131 (7.5%)	51 (9.3%)	53 (5.2%)	27 (14.4%)
Unknown	40 (2.3%)	3 (0.5%)	7 (0.7%)	30 (16.0%)
**Recommendation of GLP-1/GIP RA Medication to Person with Lipedema**				

Definitely yes	947 (54.1%)	246 (44.9%)	642 (63.4%)	59 (31.4%)
Probably yes	482 (27.6%)	177 (32.3%)	261 (25.8%)	44 (23.4%)
Unsure	183 (10.5%)	69 (12.6%)	81 (8.0%)	33 (17.6%)
Probably no	63 (3.6%)	33 (6.0%)	17 (1.7%)	13 (6.9%)
Definitely no	34 (1.9%)	20 (3.6%)	5 (0.5%)	9 (4.8%)
Unknown	40 (2.3%)	3 (0.5%)	7 (0.7%)	30 (16.0%)

NOTE: Denominator based on number of non-missing responses.

**Fig. 2:**
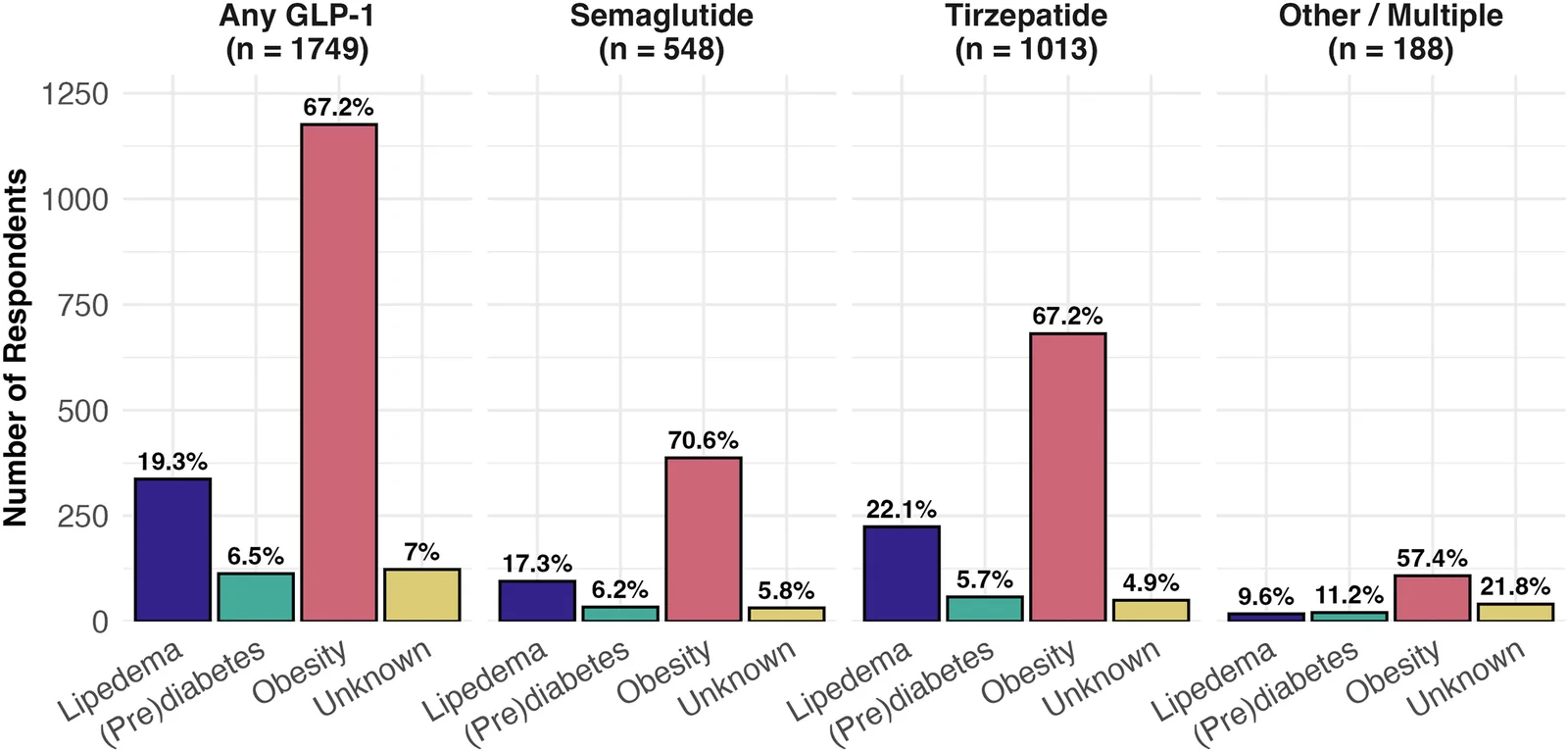
**Primary Reasons for Taking GLP-1/GIP Receptor Agonist Medications by Medication Type** Bar charts show the distribution of self-reported, primary reasons for medication use across four categories: All users combined (Any GLP-1), Semaglutide, Tirzepatide, and Other/Multiple medications. The y-axis represents the number of respondents, and percentages are displayed above each bar indicating the proportion within each medication category. Primary reasons include: Lipedema Management (Lipedema) in dark purple, Prediabetes or Diabetes Management ((Pre)diabetes) in light purple, Obesity or Weight Management (Obesity) in dark pink, and Unknown in light pink. (For interpretation of the references to color in this figure legend, the reader is referred to the Web version of this article.)

Across all incretin mimetic users, the most frequently reported primary reason for medication use was weight management (67.2%), followed by lipedema symptom management (19.3%), and then unknown (7%) or prediabetes/diabetes management (6.5%) (Fig. 2). While this trend was the same for respondents with a formal lipedema diagnosis, a slightly larger percentage (23.1%) of this group reported their primary reason for medication use was management of lipedema symptoms (Supplementary Fig. S1).

Satisfaction with the medications’ impact on general health was high: over 80% of users reported being “Very Satisfied” or “Somewhat Satisfied.” Satisfaction with the impact on lipedema symptoms was slightly lower but still high, with about 70% expressing positive satisfaction. Further, 87.6% of respondents said they would “Definitely” or “Probably” recommend incretin mimetic medications to others with lipedema (Table 2).

### 3.3 Impact on general health

To evaluate self-reported health outcomes, the PROMIS Global Health (PROMIS-10) tool was used [7,8]. Responses were analyzed for both current users, including recall of their health before use, and those who had never used GLP-1/GIP RA medications (Supplementary Table S1). We also analyzed only respondents with a formal lipedema diagnosis and saw similar results to those in the combined formal and self-diagnosed group (Supplementary Table S2). Participants who had discontinued the use of GLP-1/GIP RA medication were asked to retrospectively assess their health before and one month after discontinuing treatment (Supplementary Table S3). Across groups, results consistently reflected self-reported improvements in quality-of-life following medication use.

**Fig. 3:**
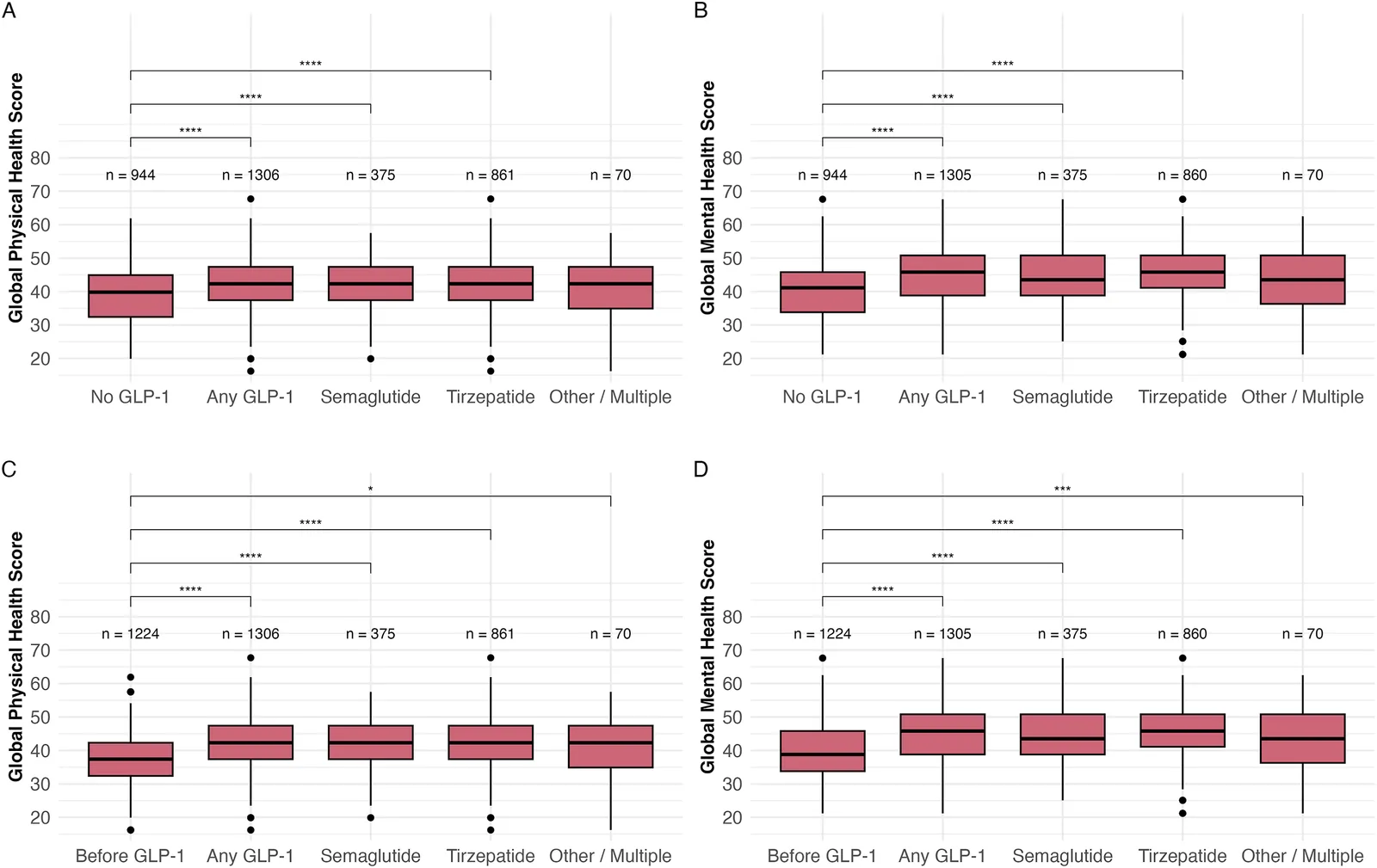
**Global Physical and Mental Health Scores Between GLP-1/GIP Receptor Agonist Users and Non-users** Box plots display Global Physical Health Scores (A, C) and Global Mental Health Scores (B, D) from the PROMIS-10 questionnaire across different GLP-1/GIP RA medication categories. Higher scores indicate better physical and mental health. Top panels (A, B) compare participants not using any GLP-1/GIP RA medication versus those currently using any GLP-1/GIP RA medication, with subgroup analysis by specific medication types (Semaglutide, Tirzepatide, Other/Multiple). Bottom panels (C, D) compare participants before using any GLP-1/GIP RA medication versus those currently using any GLP-1/GIP RA medication, with subgroup analysis by specific medication types (Semaglutide, Tirzepatide, Other/Multiple). Sample sizes (n) are indicated for each group. P-values are shown above comparisons, with statistical significance indicated by asterisks (****p < 0.0001, ***p < 0.001, *p < 0.05). Box plots show median (center line), interquartile range (box), whiskers extending to 1.5 × IQR, and outliers (dots).

PROMIS-10 provides two summary scores: Global Physical Health (GPH) and Global Mental Health (GMH) [7]. Among non-users, the average GPH and GMH scores were 39.1 and 40.8, respectively. Scores around 40 or below suggest substantially worse functioning (≈1 SD below the mean) [9]. Current users had higher scores, with tirzepatide users showing the highest self-reported scores: median GPH and GMH scores were 42.7 and 45.2, respectively (Fig. 3A and B). After adjusting for age, BMI and lipedema stage, self-reported increases in both GPH and GMH for people using either semaglutide or tirzepatide compared to non-users remained statistically significant (Supplementary Fig. S2). While users of tirzepatide had higher self-reported GPH and GMH scores after adjusting for age, BMI, lipedema stage or duration of medication, this was not statistically significant (Supplementary Fig. S3).

We also asked respondents who are currently taking incretin mimetic medications to retrospectively rate their pretreatment status using the same PROMIS-10 questions. Before starting GLP-1/GIP RA medication, people with lipedema recalled symptoms leading to GPH and GMH scores of 37.4 and 39.0, respectively. While using GLP-1/GIP RA medications, these scores rose by almost 5 points for both GPH and GMH to 42.3 and 44.9, respectively (Fig. 3C and D).

Additionally, if we separate current users of these medications into those with a formal lipedema diagnosis or those who suspect they have lipedema we see similar increases in GPH and GMH (Supplemental Figs. S4–S5). This is also true if we examine people who are using compounded vs branded medications (Supplemental Figs. S6–S7).

If we consider users who have discontinued use of GLP-1/GIP RA medication, they recalled symptoms leading to GPH and GMH scores of 38 and 40.5 before using medication. When asked to remember their symptoms approximately one month after stopping use of GLP-1/GIP RA medication, the aggregate scores rose by only 2 points for GPH to 40.2 and 3 for GMH to 43.4 (Supplementary Fig. S8)**.**

### 3.4 Impact on lipedema symptoms

To specifically evaluate self-reported impact on lipedema symptoms, we modified a subset of a questionnaire developed by Rapprich and colleagues [10]. Information was collected on participants with lipedema who have never used GLP-1/GIP RA medications, those who are currently using (Supplementary Table S4), and those who have discontinued use (Supplementary Table S5). Participants who have used these medications were also asked to recall their lipedema symptoms before using GLP-1/GIP RA medications.

**Fig. 4:**
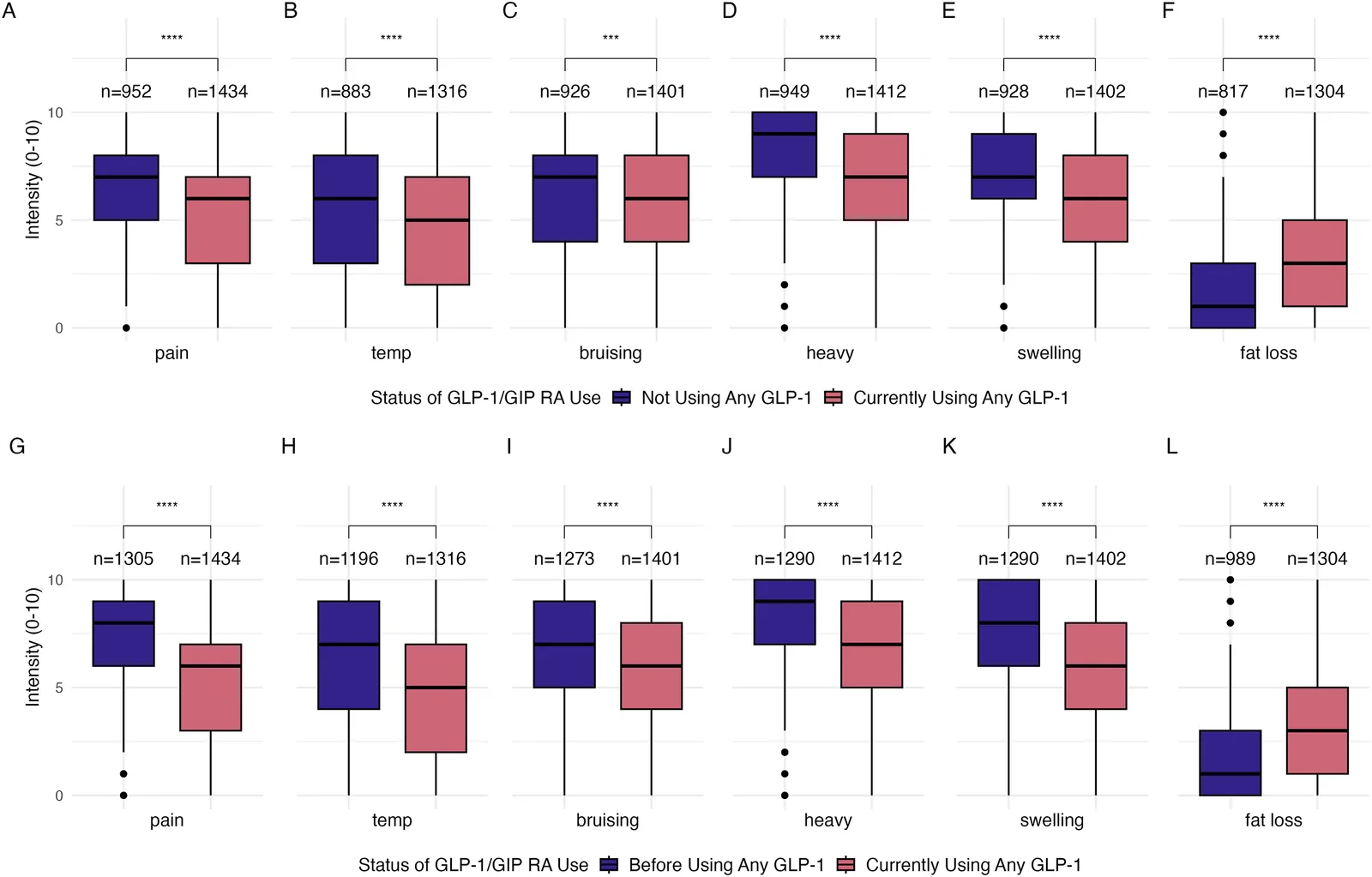
**Comparison of Lipedema Symptom Intensity Scores Between GLP-1/GIP Receptor Agonist Users and Non-users** Box plots display the distribution of self-reported symptom intensities (scale 0–10) across six dimensions: A: Do you experience pain or tenderness in the areas affected by Lipedema? (pain), B: Do you experience a feeling of warmth or coldness in the areas affected by Lipedema? (temp), C: Do you experience frequent bruising in the areas affected by Lipedema? (bruising), D: Do you experience the feeling of tired or heavy legs? (heavy), E: Do you experience any swelling in the areas affected by Lipedema? (swelling), and F: Are you able to lose fat in the areas affected by Lipedema? (fat loss). Top panels (A–F) compare participants not using any GLP-1/GIP RA medication (purple) versus those currently using any GLP-1/GIP RA medication (pink). Bottom panels (G–L) show the same symptom categories comparing participants before using any GLP-1/GIP RA medication (purple) versus currently using any GLP-1/GIP RA medication (pink). Sample sizes (n) are indicated for each group. P-values are shown above each comparison, with statistical significance indicated by asterisks (****p < 0.0001, ***p < 0.001). Box plots show median (center line), interquartile range (box), whiskers extending to 1.5 × IQR, and outliers (dots). (For interpretation of the references to color in this figure legend, the reader is referred to the Web version of this article.)

Overall, participants who used incretin mimetics self-reported improvement in lipedema symptoms. Across many of the physical dimensions assessed, participants using GLP-1/GIP RA medications reported having less intense pain or discomfort compared to either before they started using GLP-1/GIP RA medications or compared to lipedema participants who are not using GLP-1/GIP RA medications (Fig. 4). Many respondents even reported feeling like they were more able to lose fat specifically in lipedema affected regions while using GLP-1/GIP RA medications. The results were similar when people with a formal lipedema diagnosis or those self-diagnosed were analyzed (Supplementary Table S6, Supplementary Figs. S9–S10). Further, associations between GLP-1/GIP RA use and lipedema symptom scores were also consistent across strata of duration of use, medication dose, and lipedema stage (Supplementary Figs. S11–S13).

Respondents who had discontinued use of GLP-1/GIP RA medication also reported improvement of pain or discomfort while using these medications (Supplementary Fig. S14).

## 4 Discussion

This survey of 2719 individuals with lipedema represents the largest patient-reported outcome study to date examining incretin-based therapies for lipedema management. The findings reveal several important patterns that warrant detailed consideration. The use of the validated PROMIS-10 instrument allows for comparison of health outcomes against established population norms and facilitates interpretation of clinically meaningful change. The inclusion of both formally diagnosed and self-diagnosed participants reflects the real-world diagnostic challenges inherent in lipedema; importantly, sensitivity analyses limited to formally diagnosed participants yielded similar results, supporting the robustness of our findings. Additionally, the study captured a broad range of outcomes, including general health, mental health, and lipedema-specific symptoms, providing a multidimensional picture of patient experience with these medications.

### 4.1 Medication usage patterns and patient-driven adoption

Over half of respondents were current users of GLP-1/GIP RA medications. This rate is striking given that these medications are not currently approved for lipedema treatment and may not be covered by insurance for this indication. The predominance of tirzepatide use (∼60% of all users) aligns with its superior efficacy in weight reduction and metabolic improvement reported in clinical trials [12].

The finding that 67.2% of users initiated use of these medications primarily for obesity management, while only 19.3% of users initiated use specifically for lipedema management, highlights an important clinical reality. Many patients may be accessing these therapies through traditional obesity or diabetes indications, then experiencing benefits for their lipedema symptoms.

### 4.2 Clinical significance of health improvements

The self-reported PROMIS-10 global health improvements in this study represent clinically meaningful changes [13]. The 5-point increases in mean Global Physical Health (GPH) and Global Mental Health (GMH) scores among current users exceed the minimal clinically important difference typically considered significant for patient-reported outcomes [13]. Notably, current users achieved scores approaching the average range (GPH: 42.3, GMH: 44.9), while never-users remained in the impaired range (GPH: 39.1, GMH: 40.8).

These self-reported improvements are particularly remarkable given the chronic nature of lipedema and the limited therapeutic options historically available [11,14]. The self-reported gains in both physical and mental health suggest that GLP-1/GIP RA medications may address multiple dimensions of lipedema-related impairment, potentially through direct effects on symptoms as well as indirect benefits from improved mobility, reduced pain, and enhanced quality of life. An additional consideration is the unique mechanism of action of these agents in the central nervous system, particularly their role in appetite regulation [15,16]. Additionally, evidence suggests these medications may reduce what patients and clinicians increasingly refer to as “food noise”. “Food noise” is described as persistent, intrusive thoughts about food that are perceived as unwanted and dysphoric, distinguished from routine food-related thoughts by their intensity and resemblance to rumination [17]. While food noise is not considered a defining feature of lipedema, the psychological burden of the condition, including distress around eating, body image, and weight, suggests these effects may still have meaningful implications for patient well-being in this population and warrant further exploration in future studies.

### 4.3 Lipedema-specific symptom relief: implications for pathophysiology

The self-reported improvements in lipedema-specific symptoms provide valuable insights into potential mechanisms of action. Participants recalled meaningful reductions in pain, swelling, heaviness, and functional limitations – core features of lipedema that have been notoriously difficult to treat. Perhaps most intriguingly, many respondents reported enhanced ability to lose fat specifically in lipedema-affected regions, a phenomenon that challenges understanding of lipedema as a condition characterized by disproportionate fat that is resistant to diet and exercise [18,19]. This aligns with research showing that overall weight loss in individuals with lipedema results in comparable reductions in lower-body fat mass to those observed in individuals without lipedema following similar weight loss [20].

This regional fat loss capability may reflect the complex metabolic effects of incretin-based therapies, which extend beyond simple appetite suppression. A meta-analysis of randomized controlled trials of GLP-1 RAs supported use of these medications to address abnormal adipose distribution [21]. GLP-1 RAs have been shown to influence adipocyte metabolism, reduce inflammation, and improve insulin sensitivity [[22], [23], [24]]. The dual GIP/GLP-1 receptor agonists may provide additional benefits through GIP's effects on adipose tissue remodeling, metabolic function and fibrosis [[25], [26], [27]]. GIP receptor signaling specifically has been shown to activate adipocyte lipoprotein lipase (LPL) activity and to promote adipocyte differentiation through PPARγ-mediated gene regulation, effects that may complement GLP-1-mediated weight loss by altering adipose tissue lipid handling and remodeling capacity [26,28,29]. This mechanism is speculative and should not be interpreted as explaining the findings reported here, but it identifies a biologically plausible, GIP-specific pathway that merits further investigation, particularly for dual GLP-1/GIP agonists such as tirzepatide. These mechanisms could theoretically address some of the underlying pathophysiological processes in lipedema, including chronic inflammation, altered adipocyte function, metabolic dysregulation and fibrotic nodules.

### 4.4 Comparison with existing literature and treatment modalities

While controlled clinical trials of incretin-based therapies specifically for lipedema are lacking, our findings align with anecdotal reports and expert opinions [27,30,31]. The magnitude of symptom improvement reported here compares favorably with outcomes from traditional lipedema treatments such as compression therapy, though direct comparisons are limited by different outcome measures and study designs [32].

The apparent effectiveness of these medications raises questions about the role of metabolic dysfunction in lipedema pathogenesis. If incretin-based therapies are indeed providing meaningful clinical benefits beyond simple weight loss, this suggests that targeting metabolic pathways may represent a novel therapeutic approach for a condition that has historically been viewed primarily through the lens of lymphatic dysfunction and adipose tissue abnormalities.

### 4.5 Future directions

While this study provides valuable preliminary evidence, several important research directions remain. Prospective, longitudinal studies are needed to better capture treatment trajectories, assess durability of benefit, and identify potential late-emerging adverse effects. Randomized controlled trials will be essential to establish causality and quantify the magnitude of effects attributable specifically to incretin-based therapies in lipedema, including comparisons of different formulations, optimal dosing strategies, and combination approaches with established lipedema treatments such as compression therapy, manual lymphatic drainage, and liposuction.

Mechanistic studies are warranted to explore how GLP-1 and GIP receptor signaling may impact lipedema pathophysiology, particularly in relation to adipocyte function, chronic inflammation, lymphatic involvement, and regional fat distribution. Biomarker studies, imaging modalities, and tissue-level analyses could provide deeper insight into the biological mechanisms underlying the participant-reported improvements observed here.

Subgroup analyses in future studies should evaluate potential differences in treatment response by disease stage, comorbidities, and genetic background. Standardized outcome measures, including objective body composition analysis, inflammatory biomarkers, functional assessments, and validated pain measures, will be critical to enable meaningful comparisons across studies. Finally, comparing the effectiveness of incretin-based therapies with established lipedema treatments could help define their role in the broader therapeutic landscape for this underserved population.

## 5 Limitations

Several methodological limitations warrant consideration when interpreting these results. The cross-sectional design limits our ability to establish causal relationships and provides only a snapshot of treatment experiences, precluding assessment of long-term durability of benefits or late-emerging adverse effects. Reliance on self-reported outcomes introduces potential for recall bias, social desirability bias, and placebo effects. Recruitment through patient advocacy networks and social media may have introduced selection bias by preferentially capturing individuals with more severe symptoms, greater health engagement, or more positive treatment experiences, which could limit generalizability. The inclusion of both formally and self-diagnosed participants, while reflective of real-world practice, may introduce heterogeneity into the study population. We also did not have individuals report on weight-loss so cannot account for this as a factor in the data analysis. Because most of the participants in the study reported taking GLP-1/GIP RA medications for obesity, we cannot rule out that some of the self-reported general mental and physical health improvement is due to weight loss. Additionally, this study relied on self-reported perception of fat loss in lipedema-affected regions rather than objective measurement of regional body composition, therefore findings regarding regional fat loss are subjective perception rather than confirmed anatomical change. Given these constraints, findings should be considered hypothesis-generating and cannot be interpreted as evidence of a causal or mechanistic relationship between GLP-1/GIP RA use and lipedema pathophysiology. Relatedly, few participants had used GLP-1/GIP RA medications for more than two years (10.6%) or more than three years (2.5%) (Table 2), limiting our ability to assess the durability of self-reported benefits or the emergence of late effects; longer-term follow-up will be necessary to determine whether these self-reported improvements persist with continued use.

## 6 Conclusions

This study investigated the use and patient-reported outcomes of GLP-1 and GLP-1/GIP receptor agonist medications among individuals with lipedema, representing the largest survey-based examination of incretin-based therapies in this population to date. Current users self-reported clinically meaningful improvements in both global physical and mental health, as measured by 5-point gains in PROMIS-10 scores, alongside high rates of treatment satisfaction and continuation. While these findings highlight the potential of incretin-based therapies as a promising adjunct to lipedema management, the cross-sectional, survey-based design limits causal interpretation, and prospective clinical trials are urgently needed to establish evidence-based treatment protocols for this underserved population.

Key takeaways:

● GLP-1 and GLP-1/GIP RA medications are associated with clinically meaningful self-reported improvements in global physical and mental health among individuals with lipedema, with PROMIS-10 score gains exceeding the threshold for clinically significant change.
● Patient-reported improvements in lipedema-specific symptom severity, combined with high treatment satisfaction and continuation rates, suggest these therapies may offer meaningful adjunctive benefit beyond general weight management in this population.
● The current evidence base is insufficient to support definitive clinical recommendations; rigorous, prospective clinical trials are urgently needed to confirm efficacy, characterize safety, and establish standardized treatment protocols for lipedema.

## Ethics review

The research survey involving study participants received approval by the North Star Review Board (No NB500270). Informed consent was obtained from all individuals who completed the survey.

## Author contributions

**Ashok Srinivasan**: Conceptualization, Writing - Review & Editing, Supervision. **Jonathan Kartt**: Conceptualization, Methodology, Writing - Review & Editing, Supervision. **Felicitie Daftuar**: Writing - Review & Editing. **Laura D. Harmacek**: Writing - Review & Editing. **Elena Samouhos**: Writing - Review & Editing. **Stephanie Galia**: Writing - Review & Editing. **Stacey Heil**: Writing - Review & Editing. **Erin Clark**: Writing - Review & Editing. **Courtney Mascio**: Writing - Review & Editing. **Jesse C. Cochrane**: Conceptualization, Methodology, Software, Formal Analysis, Investigation, Data Curation, Writing - Original Draft, Review & Editing, Visualization.

## Poster presentation

Partial analysis of these data was presented at the annual OMA conference in April 2026.

## Declaration of artificial intelligence (AI) and AI-assisted technologies

During the preparation of this work, the author(s) used Claude for to assist in refining the manuscript's prose and to improve clarity and readability. In addition, AI-assisted tools were used to support the development and refinement of R code employed for data analysis. The author(s) reviewed and edited the output as needed and take full responsibility for the content of the published article.

## Funding

This work was supported in full by the 10.13039/100013317Lipedema Foundation.

## Declaration of competing interest

The authors declare that they have no known competing financial interests or personal relationships that could have appeared to influence the work reported in this paper.
