# Supplementary material for: GLP-1 and GLP-1/GIP receptor agonist medication use and self-reported outcomes in individuals with lipedema: Results from a large online survey
Source: Obes Pillars. 2026 Aug 14;19:100316. doi: 10.1016/j.obpill.2026.100316 (PMC13507742; doi:10.1016/j.obpill.2026.100316)
Supplement: Supplementary file 1 [file mmc1.docx]

**Supplementary Figure S1. Primary Reasons for Taking GLP-1/GIP Receptor Agonist Medications by Medication Type for Respondents with a Formal Diagnosis of Lipedema**

Bar charts show the distribution of self-reported, primary reasons for medication use across four categories for people with a formal lipedema diagnosis: All users combined (Any GLP-1), Semaglutide, Tirzepatide, and Other/Multiple medications. The y-axis represents the number of respondents, and percentages are displayed above each bar indicating the proportion within each medication category. Primary reasons include: Lipedema Management (Lipedema) in dark purple, Prediabetes or Diabetes Management ((Pre)diabetes) in light purple, Obesity or Weight Management (Obesity) in dark pink, and Unknown in light pink.

**Supplementary Figure S2. Multivariable-Adjusted Global Health and Lipedema Symptom Scores by GLP-1/GIP Receptor Agonist Use Status**

Estimated marginal means (points) with 95% confidence intervals (whiskers) for Global Physical Health, Global Mental Health, and six lipedema symptom dimensions, from linear models jointly adjusting for age, BMI, and lipedema stage, comparing No GLP-1, Semaglutide, Tirzepatide, and Other/Multiple medication groups. Sample sizes (n) are indicated for each group. Brackets show Bonferroni-adjusted p-values for each group versus No GLP-1 (****p<0.0001, ***p<0.001, **p<0.01, *p<0.05, ns = not significant), corrected across the 24 drug-comparison tests shown. Lipedema-specific symptom improvements associated with GLP-1/GIP RA use remain statistically robust after joint adjustment for these covariates. The Other/Multiple group is small (n=55-76 depending on outcome) and pharmacologically heterogeneous (dulaglutide, exenatide, lixisenatide, retatrutide, or combinations; see Section 2.3) and should be interpreted with caution.

**Supplementary Figure S3. Multivariable-Adjusted Global Health and Lipedema Symptom Scores, Semaglutide versus Tirzepatide, Adjusted for Duration of Use**

Estimated marginal means (points) with 95% confidence intervals (whiskers) for Global Physical Health, Global Mental Health, and six lipedema symptom dimensions, among current users only, from linear models jointly adjusting for age, BMI, lipedema stage, AND duration of use, comparing Semaglutide and Tirzepatide. Sample sizes (n) are indicated for each group. Bonferroni correction was applied across the 8 outcomes tested (****p<0.0001, ***p<0.001, **p<0.01, *p<0.05, ns = not significant). Point estimates favor Tirzepatide across every outcome, consistent with the unadjusted comparisons in Figure 3 and Figure 4, though only the fat-loss-ability difference remains statistically significant after joint adjustment and Bonferroni correction. Other/Multiple users were excluded from this model (see Supplementary Figure S2 notes).

**Supplementary Figure S4. Global Physical and Mental Health Scores for Participants with a Formal Lipedema Diagnosis**

Box plots display Global Physical Health Scores (A, C) and Global Mental Health Scores (B, D) from the PROMIS-10 questionnaire, restricted to participants reporting a formal lipedema diagnosis by a medical professional. Top panels (A, B) compare participants not using any GLP-1/GIP RA medication versus current users, by medication type. Bottom panels (C, D) compare participants before versus during use of GLP-1/GIP RA medication. Sample sizes (n) are indicated for each group. P-values are shown above comparisons, with statistical significance indicated by asterisks (****p<0.0001, ***p<0.001, **p<0.01). Box plots show median (center line), interquartile range (box), whiskers extending to 1.5×IQR, and outliers (dots). Results are consistent with Supplementary Figure S5 (self-reported/suspected diagnosis) and with the combined-population results in Figure 3.

**Supplementary Figure S5. Global Physical and Mental Health Scores for Participants with a Self-Reported (Suspected) Lipedema Diagnosis**

Box plots display Global Physical Health Scores (A, C) and Global Mental Health Scores (B, D) from the PROMIS-10 questionnaire, restricted to participants who suspect they have lipedema but have not received a formal diagnosis from a medical professional. Panel layout as in Supplementary Figure S4. Sample sizes (n) are indicated for each group. P-values are shown above comparisons, with statistical significance indicated by asterisks (****p<0.0001, **p<0.01, *p<0.05). Box plots show median (center line), interquartile range (box), whiskers extending to 1.5×IQR, and outliers (dots). Results are consistent with Supplementary Figure S4 (formal diagnosis) and with the combined-population results in Figure 3, indicating that diagnostic pathway does not substantially alter the observed associations.

**Supplementary Figure S6. Global Physical and Mental Health Scores for Participants Using Branded GLP-1/GIP Receptor Agonist Formulations**

Box plots display Global Physical Health Scores (A, C) and Global Mental Health Scores (B, D) from the PROMIS-10 questionnaire, restricted to participants using branded (non-compounded) GLP-1/GIP RA formulations. Panel layout as in Supplementary Figure S4. Sample sizes (n) are indicated for each group. P-values are shown above comparisons, with statistical significance indicated by asterisks (****p<0.0001, **p<0.01). Box plots show median (center line), interquartile range (box), whiskers extending to 1.5×IQR, and outliers (dots). Results are consistent with Supplementary Figure S7 (compounded formulations), indicating similar associations regardless of formulation source.

**Supplementary Figure S7. Global Physical and Mental Health Scores for Participants Using Compounded GLP-1/GIP Receptor Agonist Formulations**

Box plots display Global Physical Health Scores (A, C) and Global Mental Health Scores (B, D) from the PROMIS-10 questionnaire, restricted to participants using compounded GLP-1/GIP RA formulations. Panel layout as in Supplementary Figure S4. Sample sizes (n) are indicated for each group. P-values are shown above comparisons, with statistical significance indicated by asterisks (****p<0.0001, ***p<0.001). Box plots show median (center line), interquartile range (box), whiskers extending to 1.5×IQR, and outliers (dots). Results are consistent with Supplementary Figure S6 (branded formulations), indicating similar associations regardless of formulation source.

**Supplementary Figure S8. Global Physical and Mental Health Scores for Discontinued Users of GLP-1/GIP Receptor Agonists**

Box plots display Global Physical Health Scores (A) and Global Mental Health Scores (B) from the PROMIS-10 questionnaire across different GLP-1/GIP RA medication categories comparing participants before using any GLP-1/GIP RA medication versus immediately following discontinuation of medication. Higher scores indicate better physical and mental health. Sample sizes (n) are indicated for each group. P-values are shown above comparisons, with statistical significance indicated by asterisks (**p<0.01, *p<0.05). Box plots show median (center line), interquartile range (box), whiskers extending to 1.5×IQR, and outliers (dots).

**Supplementary Figure S9. Lipedema Symptom Intensity for Participants with a Formal Lipedema Diagnosis**

Box plots display self-reported symptom intensities (scale 0-10) across six dimensions (A-F: pain, temp, bruising, heavy, swelling, fat loss), restricted to participants reporting a formal lipedema diagnosis. Top panels (A-F) compare participants not using any GLP-1/GIP RA medication versus current users. Bottom panels (G-L) compare participants before versus during use. Sample sizes (n) are indicated for each group. P-values are shown above comparisons, with statistical significance indicated by asterisks (****p<0.0001). Box plots show median (center line), interquartile range (box), whiskers extending to 1.5×IQR, and outliers (dots). Results are consistent with Supplementary Figure S10 and with the combined-population results in Figure 4.

**Supplementary Figure S10. Lipedema Symptom Intensity for Participants with a Self-Reported (Suspected) Lipedema Diagnosis**

Box plots display self-reported symptom intensities (scale 0-10) across the same six dimensions as Supplementary Figure S9, restricted to participants who suspect they have lipedema but have not received a formal diagnosis. Panel layout as in Supplementary Figure S9. Sample sizes (n) are indicated for each group. P-values are shown above comparisons, with statistical significance indicated by asterisks (****p<0.0001). Box plots show median (center line), interquartile range (box), whiskers extending to 1.5×IQR, and outliers (dots). Results are consistent with Supplementary Figure S9 (formal diagnosis) and with the combined-population results in Figure 4, indicating that diagnostic pathway does not substantially alter the observed associations.

**Supplementary Figure S11. Effect of GLP-1/GIP Receptor Agonist Use on Lipedema Symptom Intensity, Stratified by Duration of Use**

Box plots display self-reported symptom intensities (scale 0-10) across six dimensions (pain, temp, bruising, heavy, swelling, fat loss) comparing participants not using any GLP-1/GIP RA medication (No GLP-1) versus current Semaglutide and current Tirzepatide users, stratified by duration of use: Any duration (top), less than one year (middle), and over one year (bottom). Sample sizes (n) are indicated for each group. P-values are shown above comparisons, with statistical significance indicated by asterisks (****p<0.0001, ***p<0.001, **p<0.01, *p<0.05). Box plots show median (center line), interquartile range (box), whiskers extending to 1.5×IQR, and outliers (dots). Results are consistent across duration strata, arguing against duration of use as a substantial confounder of the symptom differences reported in Figure 4.

**Supplementary Figure S12. Effect of GLP-1/GIP Receptor Agonist Use on Lipedema Symptom Intensity, Stratified by Dose Category**

Box plots display self-reported symptom intensities (scale 0-10) across the same six dimensions as Supplementary Figure S11, stratified by dose category: All doses (top), low dose (middle), and therapeutic dose (bottom). Sample sizes (n) are indicated for each group. P-values are shown above comparisons, with statistical significance indicated by asterisks (****p<0.0001, ***p<0.001, **p<0.01, *p<0.05). Box plots show median (center line), interquartile range (box), whiskers extending to 1.5×IQR, and outliers (dots). Results are consistent across dose strata, arguing against dose as a substantial confounder of the symptom differences reported in Figure 4.

**Supplementary Figure S13. Effect of GLP-1/GIP Receptor Agonist Use on Lipedema Symptom Intensity, Stratified by Lipedema Stage**

Box plots display self-reported symptom intensities (scale 0-10) across the same six dimensions as Supplementary Figure S11, stratified by lipedema stage (Stage 1-4, lipo-lymphedema). Sample sizes (n) are indicated for each group and are small for Stage 1 (n=25-30 per medication group) and Stage 4 (n=40-45 per medication group); results in these strata should be interpreted with appropriate caution. P-values are shown above comparisons, with statistical significance indicated by asterisks (****p<0.0001, ***p<0.001, **p<0.01, *p<0.05). Box plots show median (center line), interquartile range (box), whiskers extending to 1.5×IQR, and outliers (dots). Results are broadly consistent across disease stage, arguing against stage as a substantial confounder of the symptom differences reported in Figure 4.

**Supplementary Figure S14. Comparison of Lipedema Symptom Intensity Scores for Discontinued Users of GLP-1/GIP Receptor Agonists**

Box plots display the distribution of self-reported symptom intensities (scale 0-10) across six dimensions comparing participants before using any GLP-1/GIP RA medication (purple) versus immediately following discontinuation of medication (pink): A: Do you experience pain or tenderness in the areas affected by Lipedema? (pain), B: Do you experience a feeling of warmth or coldness in the areas affected by Lipedema? (temp), C: Do you experience frequent bruising in the areas affected by Lipedema? (bruising), D: Do you experience the feeling of tired or heavy legs? (heavy), E: Do you experience any swelling in the areas affected by Lipedema? (swelling), and F: Are you able to lose fat in the areas affected by Lipedema? (fat). Sample sizes (n) are indicated for each group. P-values are shown above each comparison, with statistical significance indicated by asterisks (****p<0.0001, ***p<0.001, **p<0.01). Box plots show median (center line), interquartile range (box), whiskers extending to 1.5×IQR, and outliers (dots).

**Supplementary Table 1: PROMIS-10 Assessment of Current GLP-1/GIP Receptor Agonist Medication Users and Non-Users**

This table presents patient-reported outcome measures from the PROMIS-10 (Patient-Reported Outcomes Measurement Information System) questionnaire comparing participants by GLP-1/GIP RA use status: No GLP-1 (n=970), Before GLP-1 (retrospective assessment, n=1491), Any GLP-1 (current users, n=1491), Semaglutide (n=428), Tirzepatide (n=969), and Other/Multiple medications (n=94). Data are presented as frequencies with percentages for categorical variables and means with standard deviations (SD), medians, interquartile ranges (Q1 - Q3), and minimum-maximum (Min - Max) ranges for continuous variables. The assessment includes self-reported ratings of general health, quality of life, physical health, mental health, social activities and relationships, ability to carry out social activities and roles, physical activities, emotional problems (anxiety/depression/irritability), fatigue, and pain interference. Higher scores on the PROMIS Global Physical Health (GPH) and Global Mental Health (GMH) scales indicate better health outcomes.

**Supplementary Table 2: PROMIS-10 Assessment of Current GLP-1/GIP Receptor Agonist Medication Users and Non-Users with a Formal Lipedema Diagnosis**

This table presents patient-reported outcome measures from the PROMIS-10 (Patient-Reported Outcomes Measurement Information System) questionnaire comparing participants with a formal Lipedema diagnosis by GLP-1/GIP RA use status: No GLP-1 (n=723), Before GLP-1 (retrospective assessment, n=1180), Any GLP-1 (current users, n=1180), Semaglutide (n=330), Tirzepatide (n=771), and Other/Multiple medications (n=79). Data are presented as frequencies with percentages for categorical variables and means with standard deviations (SD), medians, interquartile ranges (Q1 - Q3), and minimum-maximum (Min - Max) ranges for continuous variables. The assessment includes self-reported ratings of general health, quality of life, physical health, mental health, social activities and relationships, ability to carry out social activities and roles, physical activities, emotional problems (anxiety/depression/irritability), fatigue, and pain interference. Higher scores on the PROMIS Global Physical Health (GPH) and Global Mental Health (GMH) scales indicate better health outcomes.

**Supplementary Table 3: PROMIS-10 Assessment of Discontinued GLP-1/GIP Receptor Agonist Medication Users Before and Immediately After Treatment**

This table presents patient-reported outcome measures from the PROMIS-10 (Patient-Reported Outcomes Measurement Information System) questionnaire comparing participants by GLP-1/GIP RA use status: Before GLP-1 (retrospective assessment, n=258), and immediately following discontinuing the use of Any GLP-1 (retrospective assessment of discontinued users following cessation of treatment, n=258), Semaglutide (n=120), Tirzepatide (n=44), and Other/Multiple medications (n=94). Data are presented as frequencies with percentages for categorical variables and means with standard deviations (SD), medians, interquartile ranges (Q1 - Q3), and minimum-maximum (Min - Max) ranges for continuous variables. The assessment includes self-reported ratings of general health, quality of life, physical health, mental health, social activities and relationships, ability to carry out social activities and roles, physical activities, emotional problems (anxiety/depression/irritability), fatigue, and pain interference. Higher scores on the PROMIS Global Physical Health (GPH) and Global Mental Health (GMH) scales indicate better health outcomes.

**Supplementary Table 4: Effect of GLP-1 on Lipedema Symptoms of Current GLP-1/GIP Receptor Agonist Users and Non-Users**

This table presents detailed symptom severity scores (0-10 scale) for Lipedema-specific symptoms comparing participants by GLP-1/GIP receptor agonist use status: No GLP-1 (n=970), Before GLP-1 (retrospective assessment of current users, n=1491), Any GLP-1 (current users, n=1491), Semaglutide (n=428), Tirzepatide (n=969), and Other/Multiple medications (n=94). Data are presented as medians, interquartile ranges (Q1 - Q3), and minimum-maximum (Min - Max) ranges for each symptom domain. Symptoms assessed include pain/tenderness, warmth/coldness sensation, bruising, swelling, tired/heavy legs, nodules/lipomas, tissue fibrosis, fat loss ability, functional limitations in daily tasks, forgetfulness/cognitive issues, and difficulty focusing/maintaining attention. Higher scores indicate more severe symptoms, except for fat loss ability where higher scores indicate better ability to lose fat in affected areas.

**Supplementary Table 5: Effect of GLP-1 on Lipedema Symptoms of Discontinued GLP-1/GIP Receptor Agonist Users Before and Immediately After Treatment**

This table presents detailed symptom severity scores (0-10 scale) for Lipedema-specific symptoms comparing participants by GLP-1/GIP receptor agonist use status: Before GLP-1 (retrospective assessment, n=258), and immediately following discontinuing the use of Any GLP-1 (retrospective assessment of discontinued users following cessation of treatment, n=258), Semaglutide (n=120), Tirzepatide (n=44), and Other/Multiple medications (n=94). Data are presented as medians, interquartile ranges (Q1 - Q3), and minimum-maximum (Min - Max) ranges for each symptom domain. Symptoms assessed include pain/tenderness, warmth/coldness sensation, bruising, swelling, tired/heavy legs, nodules/lipomas, tissue fibrosis, fat loss ability, functional limitations in daily tasks, forgetfulness/cognitive issues, and difficulty focusing/maintaining attention. Higher scores indicate more severe symptoms, except for fat loss ability where higher scores indicate better ability to lose fat in affected areas.

**Supplementary Table 6: Effect of GLP-1 on Lipedema Symptoms of Current GLP-1/GIP Receptor Agonist Users and Non-Users with a Formal Lipedema Diagnosis**

This table presents detailed symptom severity scores (0-10 scale) for Lipedema-specific symptoms comparing participants by GLP-1/GIP receptor agonist use status: No GLP-1 (n=723), Before GLP-1 (retrospective assessment of current users, n=1180), Any GLP-1 (current users, n=1180), Semaglutide (n=330), Tirzepatide (n=771), and Other/Multiple medications (n=79). Data are presented as medians, interquartile ranges (Q1 - Q3), and minimum-maximum (Min - Max) ranges for each symptom domain. Symptoms assessed include pain/tenderness, warmth/coldness sensation, bruising, swelling, tired/heavy legs, nodules/lipomas, tissue fibrosis, fat loss ability, functional limitations in daily tasks, forgetfulness/cognitive issues, and difficulty focusing/maintaining attention. Higher scores indicate more severe symptoms, except for fat loss ability where higher scores indicate better ability to lose fat in affected areas.
